# Supplementary material for: Changing risk factors for placental abruption: A case crossover study using routinely collected data from Finland, Malta and Aberdeen
Source: PLoS One. 2020 Jun 11;15(6):e0233641. doi: 10.1371/journal.pone.0233641 (PMC7289359; doi:10.1371/journal.pone.0233641)
Supplement: S1 Checklist — (DOC) [file pone.0233641.s001.doc]

STROBE Statement—checklist of items that should be included in reports of observational studies

|  | | | Item No | Recommendation | Page Reported | |  |  |
| --- | --- | --- | --- | --- | --- | --- | --- | --- |
| **Title and abstract** | | | 1 | (*a*) Indicate the study’s design with a commonly used term in the title or the abstract | Title page; page 3 line 52 | |  |  |
|  |  |  |  | (*b*) Provide in the abstract an informative and balanced summary of what was done and what was found | Page 3 | |  |  |
| Introduction | | | | |  | |  |  |
| Background/rationale | | | 2 | Explain the scientific background and rationale for the investigation being reported | Page 4 lines 69 - 94 | |  |  |
| Objectives | | | 3 | State specific objectives, including any prespecified hypotheses | Page 5 lines 95 - 98 | |  |  |
| Methods | | | | |  | |  |  |
| Study design | | | 4 | Present key elements of study design early in the paper | Page 5; lines 100 -101 | |  |  |
| Setting | | | 5 | Describe the setting, locations, and relevant dates, including periods of recruitment, exposure, follow-up, and data collection | Page 5; lines 102 - 112 | |  |  |
| Participants | | | 6 | (*a*) *Cohort study*—Give the eligibility criteria, and the sources and methods of selection of participants. Describe methods of follow-up  *Case-control study*—Give the eligibility criteria, and the sources and methods of case ascertainment and control selection. Give the rationale for the choice of cases and controls  *Cross-sectional study*—Give the eligibility criteria, and the sources and methods of selection of participants | Page 6 lines 118 - 122 | |  |  |
|  |  |  |  | (*b*) *Cohort study*—For matched studies, give matching criteria and number of exposed and unexposed  *Case-control study*—For matched studies, give matching criteria and the number of controls per case | Page 6; lines 122 - 124 | |  |  |
| Variables | | | 7 | Clearly define all outcomes, exposures, predictors, potential confounders, and effect modifiers. Give diagnostic criteria, if applicable | Page 6 -7; Lines 125 - 144 | |  |  |
| Data sources/ measurement | | | 8* | For each variable of interest, give sources of data and details of methods of assessment (measurement). Describe comparability of assessment methods if there is more than one group | Page 6 – 7  Lines 102 - 144 | |  |  |
| Bias | | | 9 | Describe any efforts to address potential sources of bias | Study design | |  |  |
| Study size | | | 10 | Explain how the study size was arrived at | Figure 1 | |  |  |
| Quantitative variables | | | 11 | Explain how quantitative variables were handled in the analyses. If applicable, describe which groupings were chosen and why | Page 6 -7; lines 125 - 144 | |  |  |
| Statistical methods | | | 12 | (*a*) Describe all statistical methods, including those used to control for confounding | Page 7; lines 145 - 158 | |  |  |
|  |  |  |  | (*b*) Describe any methods used to examine subgroups and interactions | Not applicable | |  |  |
|  |  |  |  | (*c*) Explain how missing data were addressed | Page 7; Line 159 | |  |  |
|  |  |  |  | (*d*) *Cohort study*—If applicable, explain how loss to follow-up was addressed  *Case-control study*—If applicable, explain how matching of cases and controls was addressed  *Cross-sectional study*—If applicable, describe analytical methods taking account of sampling strategy | Page 7; Line 153 | |  |  |
|  |  |  |  | (*e*) Describe any sensitivity analyses |  | |  |  |
| Results | | | | | |  | | |
| Participants | 13* | (a) Report numbers of individuals at each stage of study—eg numbers potentially eligible, examined for eligibility, confirmed eligible, included in the study, completing follow-up, and analysed | | | | Figure 1 | | |
|  |  | (b) Give reasons for non-participation at each stage | | | | Figure 1 | | |
|  |  | (c) Consider use of a flow diagram | | | | Figure 1 | | |
| Descriptive data | 14* | (a) Give characteristics of study participants (eg demographic, clinical, social) and information on exposures and potential confounders | | | | Table 1 | | |
|  |  | (b) Indicate number of participants with missing data for each variable of interest | | | | Table 1 | | |
|  |  | (c) *Cohort study*—Summarise follow-up time (eg, average and total amount) | | | | Not applicable | | |
| Outcome data | 15* | *Cohort study*—Report numbers of outcome events or summary measures over time | | | |  | | |
|  |  | *Case-control study—*Report numbers in each exposure category, or summary measures of exposure | | | | *Table 1* | | |
|  |  | *Cross-sectional study—*Report numbers of outcome events or summary measures | | | |  | | |
| Main results | 16 | (*a*) Give unadjusted estimates and, if applicable, confounder-adjusted estimates and their precision (eg, 95% confidence interval). Make clear which confounders were adjusted for and why they were included | | | | Table 2 | | |
|  |  | (*b*) Report category boundaries when continuous variables were categorized | | | | Table 1 | | |
|  |  | (*c*) If relevant, consider translating estimates of relative risk into absolute risk for a meaningful time period | | | | N/A | | |
| Other analyses | 17 | Report other analyses done—eg analyses of subgroups and interactions, and sensitivity analyses | | | | N/A | | |
| Discussion | | | | | |  | | |
| Key results | 18 | Summarise key results with reference to study objectives | | | | Page 9 lines 188 -194 | | |
| Limitations | 19 | Discuss limitations of the study, taking into account sources of potential bias or imprecision. Discuss both direction and magnitude of any potential bias | | | | Page 11 – 12 lines 249 - 276 | | |
| Interpretation | 20 | Give a cautious overall interpretation of results considering objectives, limitations, multiplicity of analyses, results from similar studies, and other relevant evidence | | | | Page 12-14 lines 277 - 308 | | |
| Generalisability | 21 | Discuss the generalisability (external validity) of the study results | | | | Page 12; Lines 276 -278 | | |
| Other information | | | | | |  | | |
| Funding | 22 | Give the source of funding and the role of the funders for the present study and, if applicable, for the original study on which the present article is based | | | | Page 15; line 340 -341 | | |
