# Supplementary material for: Changing risk factors for placental abruption: A case crossover study using routinely collected data from Finland, Malta and Aberdeen
Source: PLoS One. 2020 Jun 11;15(6):e0233641. doi: 10.1371/journal.pone.0233641 (PMC7289359; doi:10.1371/journal.pone.0233641)
Supplement: S1 Table — (DOCX) [file pone.0233641.s002.docx]

| **Outcome** | **Placental abruption (n=1552, 0.3%)**  **N (%)** | **No abruption (n=550650, 99.7%)**  **N (%)** | **P value** |
| --- | --- | --- | --- |
| mode of delivery |  |  | <0.001 |
| Vaginal | 240 (15.5) | 398208 (72.3) |  |
| instrumental | 61 (3.9) | 60784 (11) |  |
| CS | 1249 (80.5) | 90258 (16.4) |  |
| Missing | 2 (0.1) | 1400 (0.3) |  |
| outcome |  |  | <0.001 |
| Livebirth | 1335 (86) | 548106 (99.5) |  |
| stillbirth | 217 (14) | 2542 (0.5) |  |
| missing | 0 (0) | 2 (0) |  |
| gestational weeks at delivery |  |  | <0.001 |
| Term | 787 (50.7) | 520207 (94.5) |  |
| moderate preterm | 158 (10.2) | 2344 (4) |  |
| very preterm | 198 (12.7) | 3080 (0.6) |  |
| extreme preterm | 404 (26) | 21806 (4) |  |
| Missing | 5 (0.3) | 3213 (0.6) |  |
| birthweight |  |  | <0.001 |
| mean | 2558.88 | 3462.14 |  |
| Normal weight | 884 (57) | 528518 (95.9) |  |
| low birthweight | 399 (25.7) | 16350 (3) |  |
| very low birthweight | 133 (8.6) | 1778 (0.3) |  |
| extreme low birthweight | 130 (8.4) | 2165 (0.4) |  |
| missing | 6 (0.4) | 2072 (0.4) |  |
| IUGR |  |  | <0.001 |
| No | 1396 (89.8) | 543394 (98.7) |  |
| yes | 43 (2.8) | 6338 (1.2) |  |
| missing | 116 (7.5) | 918 (0.2) |  |

Table S1: Comparison of perinatal outcomes of the first pregnancy with and without placental abruption
