# Supplementary material for: Changing risk factors for placental abruption: A case crossover study using routinely collected data from Finland, Malta and Aberdeen
Source: PLoS One. 2020 Jun 11;15(6):e0233641. doi: 10.1371/journal.pone.0233641 (PMC7289359; doi:10.1371/journal.pone.0233641)
Supplement: S2 Table — (DOCX) [file pone.0233641.s003.docx]

Supplementary Table S2- Comparison of perinatal outcomes of 2nd pregnancy with and without placental abruption

| **Outcome** | **Placental abruption**  **N (%)** | **Control**  **N (%)** | **P value** |
| --- | --- | --- | --- |
| mode of delivery |  |  | <0.001 |
| Vaginal | 259 (16.9) | 458244 (83.2) |  |
| instrumental | 58 (3.8) | 17484 (3.2) |  |
| CS | 1214 (79.3) | 74577 (13.5) |  |
| Missing | 0 (0) | 366 (0.1) |  |
| outcome |  |  | <0.001 |
| Livebirth | 1411 (92.2) | 549260 (99.7) |  |
| stillbirth | 120 (7.8) | 1403 (0.3) |  |
| missing | 0 (0) | 8 (0) |  |
| gestational weeks at delivery |  |  | <0.001 |
| Term | 848 (55.4) | 530460 (96.3) |  |
| moderate preterm | 12 (6.7) | 1586 (0.3) |  |
| very preterm | 153 (10) | 2362 (0.4) |  |
| extreme preterm | 428 (28) | 16263 (3) |  |
| Missing | 0 (0) | 0 (0) |  |
| birthweight |  |  | <0.001 |
| mean | 2799 | 3598 |  |
| Normal weight | 1016 (66.4) | 537914(97.7) |  |
| low birthweight | 351 (22.9) | 9986 (1.8) |  |
| very low birthweight | 89 (5.8) | 1313 (0.2) |  |
| extreme low birthweight | 72 (4.7) | 1308(0.2) |  |
| Missing | 3 (0.2) | 150 (0) |  |
| IUGR |  |  | <0.001 |
| No | 1436 (93.8) | 542377 (98.5) |  |
| yes | 46 (3) | 6511 (1.2) |  |
| missing | 49 (3.2) | 1783 (0.3) |  |
