# Supplementary material for: Changing risk factors for placental abruption: A case crossover study using routinely collected data from Finland, Malta and Aberdeen
Source: PLoS One. 2020 Jun 11;15(6):e0233641. doi: 10.1371/journal.pone.0233641 (PMC7289359; doi:10.1371/journal.pone.0233641)
Supplement: S3 Table — (DOCX) [file pone.0233641.s004.docx]

| Outcome | Pregnancy 1 | | Pregnancy 2 | |
| --- | --- | --- | --- | --- |
|  | Unadjusted OR  (95% CI) | Adjusted OR  (95% CI) | Unadjusted OR (95% CI) | Adjusted OR  (95% CI) |
| Instrumental delivery | 1.67 (1.26-2.21) | 1.95 (1.36-2.80) | 5.87 (4.41-7.81) | 5.56(3.97-7.79) |
| CS delivery | 22.96 (19.99-23.37) | 24.48 (28.86-41.19) | 28.80 (25.18-32.94) | 32.71 (28.04-38.16) |
| Stillbirth | 35.05 (30.21-40.67) | 25.45 (20.93-30.96) | 33.29 (27.43-40.41) | 30.83 (24.55-38.72) |
| moderate preterm | 44.56 (37.38-53.11) | 30.74 (24.47-38.62) | 40.20 (32.55-49.69) | 31.53 (24.64-40.34) |
| very preterm | 42.49 (36.22-49.86) | 31.53 (25.99-38.25) | 40.49 (33.93-48.32) | 32.64 (26.64-40.00) |
| extreme preterm | 12.25 (10.85-13.82) | 10.89 (9.51-12.47) | 16.45 (14.63-18.49) | 13.96 (12.21-15.96) |
| low birthweight | 14.59 (12.95-16.44) | 12.70 (11.07-14.57) | 18.61 (16.46-21.04) | 15.03 (13.01-17.36) |
| very low birthweight | 44.70 (37.04-53.96) | 33.36 (26.52-41.96) | 35.89 (28.71-44.87) | 28.08 (21.72-36.29) |
| extreme low birthweight | 35.89 (29.71-43.35) | 22.80 (17.60-29.53) | 29.14 (22.81-37.24) | 20.58 (15.33-27.62) |
| IUGR | 2.65 (1.95-3.59) | 1.95 (1.36-2.79) | 2.67 (1.99-3.58) | 1.78 (1.26-2.48) |

Table S3: Unadjusted and adjusted Odds Ratios (95% Confidence Intervals) of perinatal outcomes in pregnancies 1 and 2
