# Supplementary material for: Changing risk factors for placental abruption: A case crossover study using routinely collected data from Finland, Malta and Aberdeen
Source: PLoS One. 2020 Jun 11;15(6):e0233641. doi: 10.1371/journal.pone.0233641 (PMC7289359; doi:10.1371/journal.pone.0233641)
Supplement: Attachment — Submitted filename: Response to Reviewers.docx [file pone.0233641.s005.docx]

**We thank the reviewers and the editorial board for their constructive comments and helpful suggestions. Please find below point by point responses to the reviewers’ comments in bold. The changes in the manuscript are highlighted in yellow without track changes.**

Reviewer #1:

This study is a case-crossover study design with records of the two first pregnancies from women who had PA in one pregnancy and not the other. Cases were pregnancies with abruption and matched controls were pregnancies without abruption in the same woman. A total of 2991 women were included in their study. The study is well written, and I command the authors for the study design and size of the cohort.

**We thank the reviewer for their supportive comments.**

However, I have concerns about the importance and novelty of the data since all risk factors statistically significant in the study are well known in the literature.

**The aim of the study was to look at the effect of changing some of the already known risk factors on the occurrence of placental abruption keeping the woman-based factors (eg. Genetic predisposition) constant. In fact our starting point was to identify the risk factors implicated in the literature for abruption and see what difference any change in these would make. The novelty of this analysis lies in studying the change in these risk factors and not the risk factors *per se*. We have added a few sentences in the discussion to reflect this.**

Furthermore, in my opinion, some important data is lacking which could influence the results and for example: *Week of delivery, Induction of labor,*Mode of delivery- mainly important in the second pregnancy after PA. Since previous PA is a strong and well known it is possible that an elective Cesarean delivery was performed in an earlier week and affected the occurrence of PA in the coming pregnancy. I ask the authors to add this data if possible; * IVF treatment and alcohol use- As the authors mentioned in the limitations of the study.

**Thank you for these suggestions. As we were studying the risk factors preceding placental abruption we did not include the outcomes of pregnancies affected by abruption. We have these outcome data available apart from induction of labour which has a lot of missing data and have added these in additional supplementary tables in the manuscript. We did not have data relating to IVF treatment and alcohol use in all the constituting datasets and have already mentioned this as a weakness in the discussion.**

Regarding placenta previa as a risk factor for placenta abruption, how was the diagnosis of abruption done in the cases of placenta previa? Since bleeding is a common symptom did data regarding placenta pathology is present in the registry?

**We looked into this further and found that this was driven by the way data was collected in the Finnish birth registry. In the Medical Birth Register in Finland there are two check boxes, and the birth hospitals report these diagnoses at the same time: Placenta previa; only if diagnosed during delivery and Ablatio placentae (premature detachment of placenta). We looked at the overlap in these diagnoses and on average 5 -6 cases were diagnosed as both in each year of delivery. Thus although the absolute numbers were small, the relative risk was high.**

Reviewer #2: I really enjoyed reading your paper. It is refreshing, well designed and well written.

**Many thanks for the supportive comments.**

I have some minor comments:

1. Is it any reason that you did not include birth weight or more specifically intrauterine growth restriction (IUGR) in your data?

**As stated earlier, we were interested in looking at the risk factors for placental abruption and not the outcomes of the pregnancy and therefore did not include birthweight or IUGR in the manuscript. We have now included supplementary tables with the outcomes of pregnancy.**

2. Could you please provide the numbers and percentages in your tables as well as OR, CI and P-values?

**Thank you for this suggestion and we apologise for this oversight. This has now been included.**

3. I think you should categorize maternal age into only 3 categories: <20, 20-35 and >35. This is only a suggestion; you don’t have to do that.

**Many thanks for this suggestion but we thought there would be more granularity in the data if we categorised maternal age into smaller categories and the relatively large combined dataset allowed us to do so.**

Editorial comment:

If there are ethical or legal restrictions on sharing a de-identified data set, please explain them in detail (e.g., data contain potentially identifying or sensitive patient information) and who has imposed them (e.g., an ethics committee). Please also provide contact information for a data access committee, ethics committee, or other institutional body to which data requests may be sent.

**The dataset was created from three population based international datasets and permissions obtained from governing committees for the 3 databases. Therefore permission for public access to data will need to be given by all three committees. The Finnish register data have been given for this specific study, and the data cannot be shared without authorization from the register keepers. More information on the authorization application to researchers who meet the criteria for access to confidential data can be found at**

**https://thl.fi/fi/web/thlfi-en/statistics/information-for-researchers/authorisation-application (THL).**

**Similarly data from Aberdeen can be accessed by applying to the AMND steering committee found at https://www.abdn.ac.uk/iahs/research/obsgynae/amnd/access.php**

**The data will be deposited in a University of Aberdeen repository after the manuscript is accepted for publication.**
